# A pilot study of routine immunization data quality in Bunza Local Government area: causes and possible remedies

**DOI:** 10.11604/pamj.2017.27.239.11875

**Published:** 2017-08-02

**Authors:** Semeeh Akinwale Omoleke, Menberu Getachew Tadesse

**Affiliations:** 1 World Health Organisation, Office of Country Representative, Abuja, Nigeria

**Keywords:** Data quality, routine immunisation, coverage, vaccine preventable diseases

## Abstract

**Introduction:**

As a result of poor quality administrative data for routine immunisation (RI) in Nigeria, the real coverage of RI remains unknown, constituting a setback in curtailing vaccine preventable diseases (VPDs). Consequently, the purpose of this pilot study is to identify source(s) and evaluate the magnitude of poor data quality as well as propose recommendations to address the problem.

**Methods:**

The authors conducted a cross-sectional study in which 5 out of the 22 health facilities providing routine immunization services in Bunza Local Government Area (LGA), Kebbi State, Nigeria, were selected for data quality assessment. The reported coverage of RI in August and September, 2016 was the primary element of evaluation in the selected Health Facilities (HFs). Administered questionnaires were adapted from WHO Data Quality Assurance and RI monitoring tools to generate data from the HFs, as well as standardised community survey tool for household surveys.

**Results:**

Data inconsistency was detected in 100% of the selected HFs. Maximum difference between HF monthly summary and RI registration book for penta 3 data quality report analysis was 820% and 767% in MCH Bunza and PHC Balu respectively. However, a minimum difference of 3% was observed at Loko Dispensary. Maximum difference between HF summary and RI registration for measles was 614% at MCH Bunza and 43% minimum difference at Loko. In contrast to the administrative coverage, 60-80% of the children sampled from households were either not immunised or partially immunised. Further, the main sources of poor data quality include heavy workload on RI providers, over-reliance on administrative coverage report, and lack of understanding of the significance of high data quality by RI providers.

**Conclusion:**

Substantial data discrepancies were observed in RI reports from all the Health Facilities which is indicative of poor data quality at the LGA level. Community surveys also revealed an over-reporting from administrative coverage data. Consequently, efforts should be geared towards achieving good data quality by immunisation stakeholders as it has implication on disease prevention and control efforts.

## Introduction

In the presence of effective vaccines in the national schedule, childhood deaths from vaccine-preventable diseases (VPDs), such as pneumonia, diarrhoea, and measles, accounted for about 40% of all deaths among children under-five in developing countries including Nigeria [1, 2]. Evidence from the National Immunization Coverage Survey (NICS) indicates that variations exist in routine immunization (RI) performance across the country’s zones, with the South West (76%) and South East (91%) zones showing higher RI performance, and the North West (60%) and North East (46%) showing lowest performance [2]. This disparity ultimately impacts on national RI coverage.

The low or sub-optimal RI coverage is mainly due to factors like weak demand, poor service quality, inadequate human resource and poor local leadership and accountability [1, 3]. More importantly, the reported administrative coverage is bedevilled with poor data quality in the face of the large pool of susceptible under-fives, which could lead to outbreaks of vaccine-preventable diseases (VPDs) at various administrative levels, i.e., Local Government Area (LGA) and State [2, 4]. Consequently, this could delay or reverse the gains made through supplemental immunization activities (SIAs) for diseases that are targeted for eradication and elimination such as Polio and Measles respectively [5].

According to administrative coverage reports, routine immunization showed consistent and increasing trend in Kebbi State, including Bunza Local Government Area (LGA). However, findings from community-level survey put in doubt the validity of these administrative figures, as there are disparities between these data sources. Perhaps, reported cases of VPDs, and sometimes outbreaks could be an indication of immunity gaps suggesting that the administrative coverage often reported may not be correct. Further, the surveillance and outbreak response reports in the state corroborated these findings. Similarly, national survey results following measles follow-up campaign in 2015 also revealed a great discrepancy between the reported administrative coverage and coverage surveys (104% versus 80.4%) [6]. Likewise, observations from community surveys during RI supervision by senior supervisors from the state (government and partners) showed huge difference among fully immunized children compared to the high reported administrative coverage in the same catchment health facility providing service to same settlement where surveys were conducted.

In the light of the above, World Health Organization recommended data quality checks at different levels [7]. Consequently, partners are making effort to support the government to address this issue. For instance, in Kebbi State, the attention of programme managers sand relevant stakeholders has been drawn to data quality issues during the routine technical review meetings and supportive supervision by partner agencies. Sadly, there was no formal assessment to specifically investigate the basis of the discrepancies and proffer possible remedies. Hence the need to pilot a study seeking to understand how the discrepancy originates along recording and reporting line, the extent of the discrepancy, and to identify the root cause(s) as well as recommend action points towards improving data quality. This study will also form a foundation for a more robust study, perhaps a state-wide assessment on this subject.

## Methods

A cross-sectional facility and community level study was carried out in five health facilities (HFs) and selected settlements under each facility of Bunza LGA, Kebbi state, Nigeria between August and September 2016.

### Study area

Bunza is one of the 21 LGAs in Kebbi state with a total population of 164,825 based on 2006 census population and 6,593 under-one population. The LGA shares borders with Kalgo, Arewa, Dandi and Zuru LGAs. There are 4 Traditional District Heads and 10 political wards under Bunza LGA. However, most of the wards are hard-to-reach for health services. There are 22 health facilities (HFs) in the LGA providing RI services through fixed and outreach strategies. The health facilities under study were selected based on their reported coverage of RI in August and September, 2016; the five health facilities reported maximum performance based on coverage.

### Data collection

A semi-structured questionnaire was used to collect primary and secondary data from health facilities. The questionnaire was adapted from multiple tools such as the WHO Data Quality Assurance and RI monitoring tools while important areas believed to be relevant from supervisory observations were also included. For the household survey, the standardized community survey tool was improvised by including the proposed recommendations from mothers/care takers to address the reasons mentioned for not vaccinating or defaulting from immunization sessions.

For all the sessions conducted in September 2016, Penta 3 and measles antigens were checked in the Tally sheet, RI register, HF summary and the same copy sent to the LGA were included in the data check. In addition, same summary was checked at the LGA (received from HF and sent to the state). To explore the reasons for discrepancies of the RI data across the different data tools and at different levels, RI providers and RI programme managers were asked about the reason(s), the presence of regular monitoring mechanisms and feed backs for data quality, and what to do to address the issues.

One settlement was selected from each HF catchment area and 10 under-one year children were surveyed for their immunisation status giving a total of 50 children sampled from all the settlements. Similarly, mothers/caregivers of children who did not complete their antigen for their age and/or not started at all were interviewed about the reasons for the status of immunisation and their recommendations.

The WHO Field Volunteers and Local Government Area Facilitator were trained at the LGA Level and deployed for data collection. The tools were pre-tested at one of the HFs. The data collection processes were supervised by senior personnel from a partner agency, specifically, WHO Cluster Coordinator, and the entire processes were overseen by the State Coordinator.

### Data analysis

Quantitative primary and secondary data from all sources were summarized, analysed and presented in tables and graphs while comparisons and conclusions made from summaries. Discrepancies of data across the different data tools were calculated by subtracting the values between two data tools with maximum difference taken as ‘numerator’, then the value in a data tool with minimum record taken as ‘denominator’, and then presented in percentages. Qualitative data analysis was made after developing thematic framework using responses, coded, compiled and summarized manually, then complemented with the quantitative results of primary and secondary data sources. Finally, the findings from the quantitative and qualitative data were presented in narrations and tables.

### Ethical consideration

We obtained consent from all the RI providers, mothers/care takers and RI program managers at LGA level who participated in the study, after explaining the purpose of the study to them. We also got ethical clearance from the Research and Ethical Committee of the Kebbi State Ministry of Health.

## Results

A total of five health facilities were assessed using the DQA tool, all RI providers of the respective facilities, and one Local Immunisation Officer were interviewed with a response rate of 100%. Profiling the health workers showed that 50% of them were Community Health Extension Workers and the rest were Environmental Health Technicians (akin to an Ordinary Diploma) by qualification.

### Data quality assessment results

**Figure 1:**
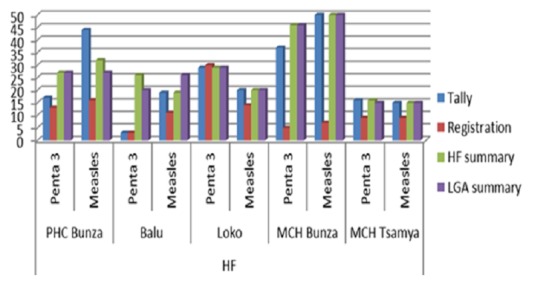
Bar chart showing data from different data tools in five health facilities and LGA summaries

From the Data Quality Assessment (DQA) at the HFs and LGA, discrepancies across the different RI data tools were observed in 100% of the HFs (Table 1). The difference was also observed in all the different data tools (tally, registration and HF summary). Maximum difference for penta 3 was observed between the HF monthly summary and RI registration book (820% and 767% difference) at MCH Bunza and PHC Balu respectively. Minimum difference was observed at Loko Dispensary (3%). For measles, maximum difference was noted between HF summary and registration at MCH Bunza (614%), and lowest at Loko Dispensary (43%) (Figure 1).

**Table 1:** Data from the different RI data tools by Health Facilities at Bunza LGA in September 2016

RI data tool	Health Facilities									
	PHC Bunza		Balu		Loko		MCH Bunza		MCH Tsamiya	
	Penta 3	Measles	Penta 3	Measles	Penta 3	Measles	Penta 3	Measles	Penta 3	Measles
Tally	17	44	3	19	29	20	37	50	16	15
Registration	13	16	3	11	30	14	5	7	9	9
HF summary	27	32	26	19	29	20	46	50	16	15
LGA summ	27	27	20	26	29	20	46	50	15	15
% (Max	108%	38%	767%	73%	3%	43%	820%	614%	78%	67%

### Reasons for data discrepancy

Interviews with RI providers on the reasons for data discrepancy include the following: non-availability of data tools at outreach sessions (poor logistics funding), no regular feedback on data quality from LGA, paucity of personnel leading to work overload (limited time available to record and complete data tools due to competing clinical responsibilities). These were the commonest reasons mentioned by 100%, 60% and 85% of the respondents respectively. Additional personnel during RI sessions (service delivery) deployed from LGA and the need to review and check data tools before sending to the next level were mentioned by RI providers as potential remedies for improving data quality.

### Community survey findings

**Figure 2:**
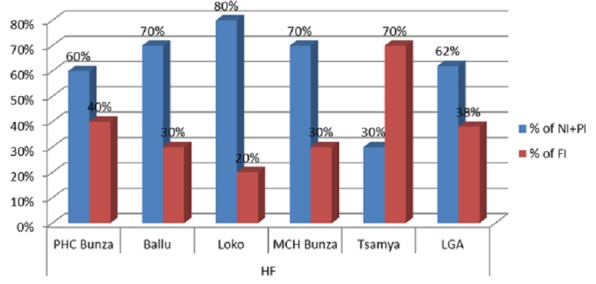
Community survey results for RI at different settlements under selected HFs at Bunza LGA, September 2016

As can be seen from the table, findings of the community survey showed only 20%-40% of children were fully immunized for their age while 60-80% of children were either not vaccinated or partially immunized. The overall coverage for the LGA appeared to be 38% fully vaccinated (Figure 2). Mothers/caregivers provided reasons for not vaccinating their children or defaulting during RI sessions. Common reasons stated by mothers were succinctly captured as thus: *“Sessions were not informed ahead of the schedule, sessions were interrupted, fear of side effects and minor complaints after injections and lack of awareness on benefits of vaccines”* were among major reasons mentioned by mothers/caregivers. They also suggested *“prior announcement and reminder for planned sessions should be made for them not to default their child from subsequent antigens administration”*.

## Discussion

Routine immunisation services are being delivered at 22 health facilities in Bunza LGA via outreach and fixed post strategies. On the average, each HF conducts 8 sessions in a month. All sessions conducted with one RI provider who is otherwise known as RI-in-charge; meaning that he or she is responsible for all RI activities at that HF. Sadly, nearly half of the RI providers (50%) are environmental health technicians. According to the national guideline in Nigeria, Environmental Health Technicians are not expected to provide RI services [8]. It should be noted that all health facilities in Kebbi State developed ‘Reaching Every Ward’ (REW) micro plan every quarter for their catchment area and also used RI data tools (RI tally, registration, child health card and summary sheets) in documenting RI sessions.

Over all, the reported RI administrative coverage was satisfactory at the LGA level, despite the fact that Bunza is one of the least performing LGAs when compared with the other LGAs in the state [9]. DQA provides specific information as to which reporting units contributes more for variation and inconsistency in the reporting system and diagnose specific weakness that, if addressed, could improve the precision and data quality [7].

According to the data analysis finding at the two reporting levels (HF and LGA), differences were observed for antigens checked (Penta 3 and Measles) in all RI data tools (tally, registration, and summary). In a data quality audit conducted in Tunisia, re-counts from immunization registration books seem to provide more accurate data [10], hence, the basis of comparison or variation using the RI register in our study. The maximum difference for penta 3 was observed between the HF monthly summary and RI registration book at MCH Bunza and Balu (820% and 767% difference) respectively. This variation is the highest as compared with similar studies conducted in Nigeria and other African countries [10–12]. Minimum difference was at Loko Dispensary where only 3% variation was observed. For measles, maximum difference was between LGA summary and registration at MCH Bunza (614%) and lowest at Loko (43%). Likewise, the maximum variation for measles was 614% which is much higher than findings from data quality assessment conducted in Mozambique, where 268% variation was reported [11]. Different assessments on precision of data reported similar finding on data inconsistencies and missed data, and our assessment showed 79% accuracy ratio at health facilities which is less than 85% reported in Tunisia [10].

The cumulative reported administrative coverage for HFs and LGA was more than 100% whereas community survey findings showed greater discrepancies where the maximum coverage was found to be 70% at Tsamiya HF and lowest 20% at Loko HF, using survey data of fully immunised children. The LGA coverage from this HFs was 38% (fully immunized for their age) and 62% were either not immunized (32%) or partially immunized (30%). This finding is consistent with regular RI supervisory findings at community level using the same approach and tools (analysis of RI abridged checklist administration). Furthermore, this finding corroborates findings from the National Primary Health Care Development Agency (NPHCDA)-supported 2015 measles mass vaccination coverage survey that reported coverage of 80.4% by history and card and 58% by card only [6].

The above findings have implication on achieving herd immunity. Herd immunity is believed to be maintained if 90-95% “true” coverage is achieved and maintained in any defined geographical area for the different RI antigens [3]. In view of above findings, the eradication and elimination goal for targeted VPDs is in doubt, if these negative findings (poor data quality) are not addressed.

Based on our finding, heavy work load on RI providers was the major contributing factor for untimely tallying and registration on the RI data tools, particularly at outreach sites, where one provider could not be able to carry all the tools to the outreach session, and strained to register the client. In addition, efforts from high level supervisors were mainly targeted at improving overall coverage rather than the consistency of data using different data tools and at different levels. Paying more attention to data quality, de-emphasising on high coverage and use of multiple data sources to validate administrative coverage could be some of the proffered solutions towards halting and reversing these ugly findings.

According to the response from the mothers/parents of unimmunized and partially immunized children on the reasons for partial or non-immunisation of children, the fact that parents were not informed and were not aware of the sessions was the leading contributing factor, indicating a weak community link to health facilities and inadequate social mobilization. This is in spite of the existence of mobilizers and VDCs as part of primary health care (PHC) structure. These parts of PHC structure, however, needs revitalisation and greater community engagement. Previous studies [1, 13–15] also corroborated these findings (weak VDC and inadequate social mobilisation).

Parents’ fear of side effects after the first or second dose of an antigen was a contributing factor for partially immunized children. The reasons that emerged from this study is in agreement with the study conducted in Sudan [14]. The Sudanese study also pointed out that lack of awareness on benefits, not informed of the sessions and providers absent at service delivery points [14]. This underscores the need to improve the knowledge, attitude, and interpersonal communication skills of RI providers.

Further, it seems that RI in-charges and LGA officials are lacking in their understanding of the relevance of ensuring good data quality as it impacts on population immunity. Therefore, it becomes imperative to educate and emphasise to RI providers and programme managers at the LGA level on the (direct and indirect) consequences of poor data quality, unreliable routine administrative coverage in prevention and control of vaccine preventable diseases. This was also emphasised in the recent (2013-2015) National Routine Immunisation Strategic Plan [15].

### Limitations of the study

The research conducted was limited to one LGA (Bunza) which may not be entirely representative of the degree of poor data quality in routine immunisation reporting in Kebbi State. However, the study provided a clue to understanding the origin of the discrepancy along the reporting chain, the magnitude of the discrepancy, the cause of the observed disparities, and the suggested evidence-based recommendations to mitigate the negative findings. Secondly, the study was limited to LGA-level data, however, it could have been more informative if state-level data were included.

## Conclusion

This study revealed a significant data discrepancy in all antigens examined at all levels of the reporting line. Differences were also observed in the community survey where majority of the sampled children were found either not immunized or partially immunized for their age, which is a sharp contrast to administrative coverage data. Lack of focus on the data quality, poor understanding of the impact of poor data quality, inadequate logistics support, especially for conduct of outreach services, and high workload on RI providers were identified as contributing factors to the huge discrepancies observed in RI data. Hence, cognizant to the prevailing situation and the findings above, recommendations are provided as follows: 1) The immediate and medium term consequences of poor data quality should be emphasized through evidence-based advocacy to Local Government Authorities, Program Managers and Health workers (RI providers) while efforts should be geared towards swiftly addressing the identified problems. 2) Data quality component in the routine supportive supervision should be at the front burner while enforcing the use of RI data tools and emphasising the relevance of high data quality. 3) Staffing to match workload should be looked into to facilitate effective data collection and reporting, especially at the facility level, and further assessment on the quality of RI personnel delivering RI services should be considered. 4) Strengthening community linkage activities and demand creation for RI services remain vital for improved RI uptake and ultimately, vaccine preventable diseases prevention. 5) In view of the magnitude and its importance, detailed analysis of the root cause(s) of data discrepancy with a wider scope is also highly recommended.

### What is known about this topic

- There is an existing protocol recommended by the World Health Organisation (WHO) on effective utilisation of data tools and review of routine immunisation data for the sole purpose of Data Quality Audits (DQA);
- The Federal Ministry of Health, Nigeria also provided guidelines on proper maintenance of logbooks at HFs and LGAs to permit higher accuracy in data reports of vaccination coverage at national level;
- Some studies have provided evidence linking poor data quality to low RI coverage but no emphasis has been placed on the exact cause(s) and true extent of poor data quality in Northern Nigeria.

### What this study adds

- In Northern Nigeria, this pilot study is the first attempt known to the authors that specifically investigates the sources of poor RI data quality, the magnitude and proffer recommendations to alleviate this problem at the LGA level;
- The study reveals that most of the discrepancies are at the health facility level usually between the tally sheet data, the registration books and the health facilities’ summaries;
- The paper unearths the main root causes of poor data quality in this environment, namely, heavy workload (paucity of trained personnel), poor utilisation of data tool and lack of understanding of the significance of quality data, inadequate logistics support (inadequate transport for outreach services and non-availability of some data tools); The paper emphasised the dire need to improve data quality as an approach to effective disease prevention and control, especially vaccine-preventable diseases.

## Competing interests

The authors declare no competing interest.
